# Sacubitrilat exerts potent antiarrhythmic effects through activation of natriuretic peptide receptor-C

**DOI:** 10.1093/eschf/xvag253

**Published:** 2026-10-16

**Authors:** Manuel J Vogel, Luca F Gerres, Jonas Herting, Michael Kohlhaas, Lisa Krebes, Michaela Kuhn, Sabine Rebs, Katrin Streckfuss-Bömeke, Thomas Müller, Stefan Frantz, Christoph Maack, Thomas H Fischer

**Affiliations:** Department of Internal Medicine I, University Clinic Würzburg, Oberdürrbacherstraße 6, Würzburg D-97080, Germany; Comprehensive Heart Failure Center, University Clinic Würzburg, Am Schwarzenberg 15, Würzburg D-97078, Germany; Department of Internal Medicine I, University Clinic Würzburg, Oberdürrbacherstraße 6, Würzburg D-97080, Germany; Comprehensive Heart Failure Center, University Clinic Würzburg, Am Schwarzenberg 15, Würzburg D-97078, Germany; Department of Internal Medicine I, University Clinic Würzburg, Oberdürrbacherstraße 6, Würzburg D-97080, Germany; Comprehensive Heart Failure Center, University Clinic Würzburg, Am Schwarzenberg 15, Würzburg D-97078, Germany; Comprehensive Heart Failure Center, University Clinic Würzburg, Am Schwarzenberg 15, Würzburg D-97078, Germany; Institute of Physiology, University of Würzburg, Röntgenring 9, Würzburg D-97070, Germany; Institute of Physiology, University of Würzburg, Röntgenring 9, Würzburg D-97070, Germany; Institute of Pharmacology and Toxicology, University of Würzburg, Versbacher Straße 9, Würzburg D-97078, Germany; Institute of Pharmacology and Toxicology, University of Würzburg, Versbacher Straße 9, Würzburg D-97078, Germany; Medical Clinic I, Cardiology and Angiology, Justus-Liebig-University Gießen, Rudolf-Buchheim-Straße 8, Gießen D-35392, Germany; Molecular Plant Physiology and Biophysics—Botanics I, University of Würzburg, Julius von Sachs Platz 2, Würzburg D-97072, Germany; Department of Internal Medicine I, University Clinic Würzburg, Oberdürrbacherstraße 6, Würzburg D-97080, Germany; Comprehensive Heart Failure Center, University Clinic Würzburg, Am Schwarzenberg 15, Würzburg D-97078, Germany; Department of Internal Medicine I, University Clinic Würzburg, Oberdürrbacherstraße 6, Würzburg D-97080, Germany

**Keywords:** Sacubitril, Entresto, Natriuretic peptides, NPR-C, Ventricular arrhythmias

## Abstract

**Introduction:**

Sacubitril, a neprilysin inhibitor used in the treatment of heart failure, has been shown to exert direct antiarrhythmic effects on ventricular cardiomyocytes. However, the underlying mechanisms and molecular targets remain to be elucidated. This study, therefore, aimed at investigating the direct antiarrhythmic properties of sacubitril and exploring a potential involvement of natriuretic peptides in the context of arrhythmia-induced cardiomyopathy (AIC).

**Methods and Results:**

Diastolic sarcoplasmic reticulum (SR) Ca^2+^ leak was induced in isolated murine ventricular cardiomyocytes by enhancing late sodium current with ATX-II, and in human induced pluripotent stem cell-derived ventricular cardiomyocytes (iPSC-CM) by 24-h tachyarrhythmic stimulation. Diastolic Ca^2+^ leak and Ca^2+^ transient kinetics were quantified using confocal and epifluorescence microscopy, while pharmacological tools (CNP, ANP, NPR-C agonist cANP_4-23_, and NPR-C blocker osteocrin) dissected receptor involvement. *In silico* modelling explored potential binding sites of sacubitrilat to NPR-C. CNP, but not ANP, reversed the increases in Ca^2+^ spark frequency induced by ATX-II in murine ventricular cardiomyocytes. This effect was reproduced by selective NPR-C activation with cANP_4-23_ and abolished by NPR-C blockade with osteocrin. In a similar manner, sacubitrilat suppressed pathological SR Ca^2+^ leak in both murine and human iPSC-derived cardiomyocytes. This protection was fully lost when NPR-C was inhibited. Sacubitrilat did not cause any significant alterations in systolic Ca^2+^ release and SR Ca^2+^ load in murine ventricular cardiomyocytes. *In silico* modelling identified a plausible hydrophobic binding pocket for sacubitrilat at the NPR-C dimer, thus supporting a direct receptor interaction.

**Conclusions:**

These data indicate that activation of NPR-C is both necessary and sufficient for the potent antiarrhythmic actions of sacubitrilat, mediating a selective reduction of arrhythmogenic SR Ca^2+^ leak in murine ventricular cardiomyocytes as well as in a cellular model of human AIC in iPSC-CM. NPR-C therefore emerges as a promising molecular target for the development of novel antiarrhythmic therapies suitable for patients with heart failure and arrhythmia-induced cardiomyopathy.

## Graphical Abstract

**Graphical Abstract:**
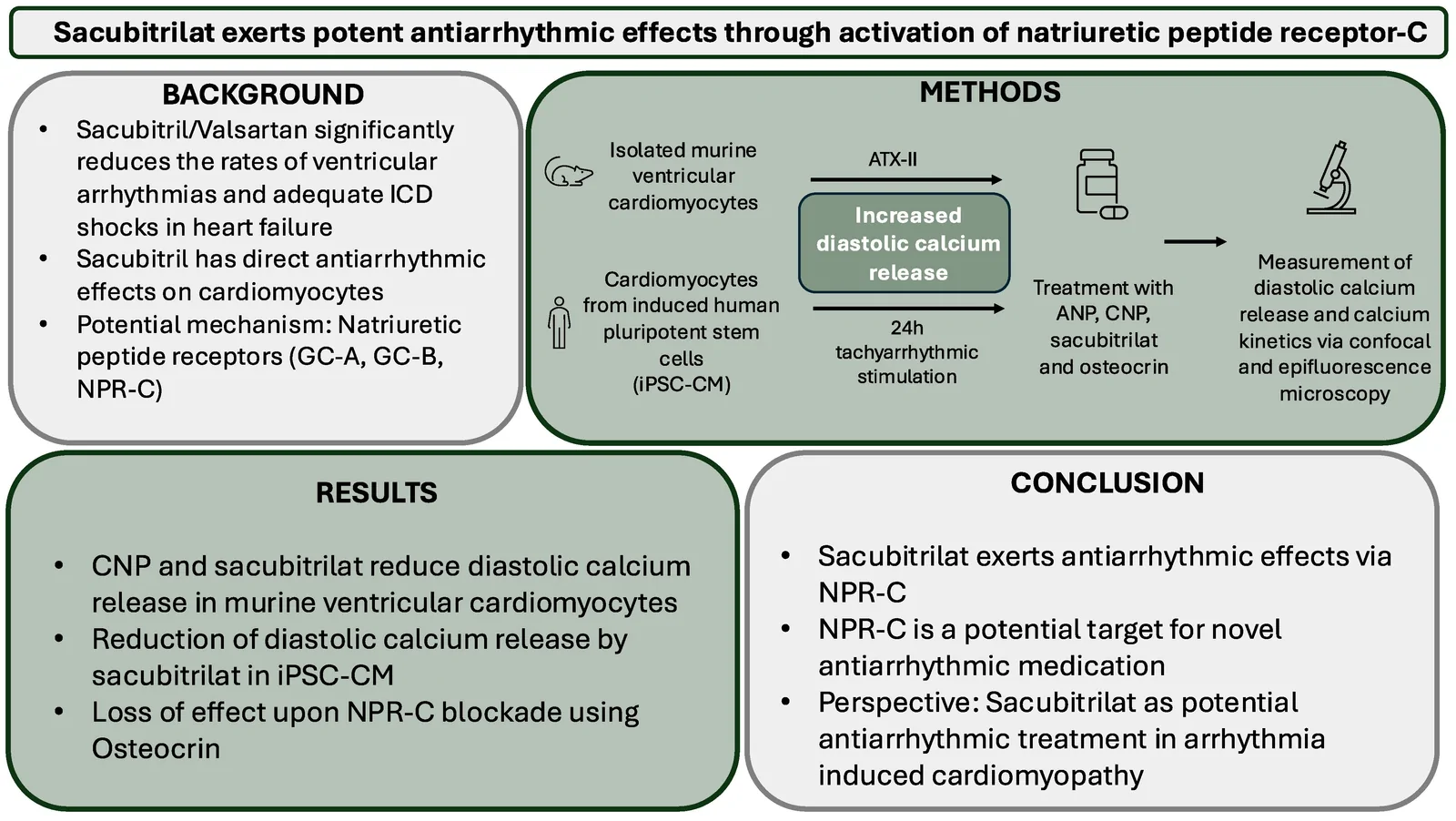

## Introduction

Arrhythmia-induced cardiomyopathy (AIC) is a complex condition characterized by the progressive deterioration of cardiac function and electrical instability due to irregular atrial or ventricular excitation. A key characteristic of AIC is the partial or complete recovery of left ventricular systolic dysfunction following the restoration of normal sinus rhythm. The pathophysiology of AIC is multifactorial, involving a combination of haemodynamic stress, electrical and structural cardiomyocyte remodelling and altered calcium (Ca^2+^) handling as well as increased sympathetic nervous system activation.^1^ Over time, these factors contribute to worsening ventricular function and the development of life-threatening arrhythmias.^1^ Therefore, antiarrhythmic therapy is a cornerstone of AIC treatment.

In AIC and other forms of heart failure, dysregulation of intracellular cardiomyocyte Ca^2+^ handling plays a crucial role in arrhythmogenesis. Phosphorylation of the ryanodine receptor type 2 (RyR2) by CaMKII (Ca^2+^/calmodulin-dependent protein kinase II) and protein kinase A (PKA) and other post-translational modifications disrupt its normal closure during diastole, causing abnormal, localized Ca^2+^ release events, known as Ca^2+^ sparks.^2–4^ The diastolic Ca^2+^ release impairs the heart's electrical stability, since a significant amount of Ca^2+^ is exchanged for Na^+^ ions via the sarcolemmal Na^+^/Ca^2+^ exchanger (NCX; 3 Na^+^ for one Ca^2+^).^3,4^ This leads to a net inward current of positive ions, which is a known trigger for ventricular arrhythmias.^3^ In this study, diastolic Ca^2+^ leak and late sodium current (late *I*_Na_) is experimentally induced by (i) administration of ATX-II (murine ventricular cardiomyocytes), which potently enhances late *I*_Na_ and (ii) 24-h tachyarrhythmic stimulation of cardiomyocytes derived from human-induced pluripotent stem cells.^5^ Late *I*_Na_ is known to be elevated in AIC and other types of heart failure and reduces the net repolarization current, leads to a prolonged action potential duration and facilitates early and delayed afterdepolarizations.^4–8^ Furthermore, it was shown that late *I*_Na_ is closely linked to dysregulated Ca^2+^ homeostasis, as it activates CaMKII and PKA (through enhanced cAMP) and thus contributes to proarrhythmogenic diastolic Ca^2+^ leak.^5^ Through these mechanisms, late *I*_Na_ promotes ventricular arrhythmias.^8^

Clinical research has shown that sacubitril/valsartan exerts potent antiarrhythmic effects by reducing ventricular arrhythmias and the number of appropriate implantable cardioverter defibrillator (ICD) shocks in patients with heart failure with reduced ejection fraction (HFrEF).^9,10^ The antiarrhythmic effect may be attributed to several potential mechanisms, including reverse cardiac remodelling, anti-inflammatory, anti-fibrotic, and anti-hypertrophic effects, as well as the modulation of the sympathetic nervous system.^11–15^ Moreover, a direct influence on the electrical stability of the cardiomyocytes might also play a role in the antiarrhythmic properties of sacubitrilat (sac).

We previously observed that sacubitrilat exerts a direct antiarrhythmic effect on murine and human ventricular cardiomyocytes *in vitro* through mitigation of Ca^2+^ handling abnormalities.^16^ However, the underlying mechanisms are currently still unresolved. Sacubitrilat is known to potently inhibit neprilysin, which is responsible for the enzymatic degradation of natriuretic peptides. Consequently, these peptides (atrial-, brain-, C-type natriuretic peptide; ANP, BNP, CNP) and their specific receptors (guanylyl cyclase receptors A and B, GC-A, GC-B; and natriuretic peptide receptor C, NPR-C) may be involved in the antiarrhythmic effect of sacubitrilat. ANP and BNP, primarily secreted from cardiomyocytes in response to mechanical stretch, bind to GC-A, while CNP, mainly released by endothelial cells and fibroblasts, binds to GC-B, mainly exerting local auto/paracrine functions.^17,18^ The activation of both GC-A and GC-B has been shown to increase intracellular cGMP levels, thereby exerting effects primarily through cGMP-dependent modulation of kinases (cGKI) and phosphodiesterases (PDEs 2A and 3).^17^ All three natriuretic peptides interact with NPR-C.^17^ It is noteworthy that NPR-C, which was previously regarded as a mere clearance receptor, has the ability to couple with inhibitory G-proteins (G_i_), which leads to the inhibition of adenylyl cyclase and thus lowers intracellular levels of cyclic adenosine monophosphate.^19^

Given the limited availability of effective and well-tolerated antiarrhythmic therapies for patients with HFrEF, including AIC, identifying novel molecular targets is of paramount importance. This study, therefore, aimed at investigating the direct antiarrhythmic properties of sacubitrilat and exploring a potential involvement of natriuretic peptides in the context of AIC.

## Methods

### Isolation of murine cardiomyocytes

The animal study was conducted in accordance with local legal regulations, and procedures were performed in accordance with the NIH guidelines (guide for the care and use of laboratory animals) and the guidelines from Directive 2010/63/EU of the European Parliament on the protection of animals used for scientiﬁc purposes. All animal experiments were approved by the local governmental authorities (approval no. RUF 55.2.2-2532-2-659 & 1755). C57B6N-wildtype-mice were pretreated with heparin (500 IU/kg) and buprenoved (0.1 mg/kg) by an intraperitoneal injection prior to isolation to ensure proper isolation of ventricular cardiomyocytes. Murine hearts were explanted after anaesthesia with isoﬂurane (3%) and retrogradely perfused in a Langendorff apparatus with an initially Ca^2+^-free solution containing (in mmol/L) NaCl 113, KCl 4.7, KH_2_PO_4_ 0.6, Na_2_HPO_4_×2 H_2_O 0.6, MgSO_4_×7H_2_O 1.2, NaHCO_3_ 12, KHCO_3_ 10, HEPES 10, Taurine 30, 2,3-butanedione monoxime (BDM) 10, glucose 5.5, and phenol red 0.032 (37,8°C, pH 7.4). Then, liberase 1 (final concentration 0.25 mg/ml, Roche Diagnostics, Mannheim, Germany) and trypsin (final concentration 0.14 mg/ml, Life Technologies, Carlsbad, CA, USA) as well as CaCl_2_ (final concentration 12,5 µmol/l) were added to start digestion. After stopping the digestion, the ventricular myocardium was separated from the atria and cut into small pieces. Concentration of Ca^2+^ was increased in a stepwise manner every 4 min until a targeted concentration of 1 mmol/l was reached.

### Confocal microscopy

Isolated murine cardiomyocytes were positioned on laminin (Roche, Basel Switzerland) coated measuring chambers and allowed to settle for a period of 15 min. The cells were then incubated with a Fluo-4 a.m. loading buffer (10 µmol/L; Thermo Fisher Scientific, Waltham, MA, USA) at room temperature for 15 min. Subsequently, de-esterification was conducted for 15 min at room temperature. In the control group, this was performed with a drug-free Tyrode’s solution, whereas in the treatment groups, it was conducted with a Tyrode’s solution containing the respective active agent. The experimental Tyrode’s solution contained (mmol/L): KCl 5, NaCl 130, MgCl_2_ 1, Na-HEPES 10, glucose 10, Na-pyruvate 2, ascorbic acid 0,3, CaCl_2_ 1 and the respective active agents (pH 7.4, room temperature).

The late sodium current (late I_Na_) was increased by the addition of ATX-II (5 nmol/l or 0.5 nmol/l, Alomone Labs, Jerusalem, Israel) as described previously to induce an imbalance of Na^+^-/Ca^2+^-homeostasis mimicking conditions found in heart failure.^5,20^ For the initial proof-of-concept experiments comparing the effects of ANP and CNP, cardiomyocytes were challenged with 5 nmol/L ATX-II to establish a robust arrhythmogenic phenotype. All subsequent mechanistic experiments investigating the effects of sacubitrilat and NPR-C signalling were performed using 0.5 nmol/L ATX-II to model a more moderate, enhancement of late I_Na_ as it is found in heart failure.

The corresponding active substances were added in addition to ATX-II depending on the treatment group: CNP (100 nmol/l, Bachem, Bubendorf, Switzerland), ANP (100 nmol/l, Bachem, Bubendorf, Switzerland), sacubitrilat (40 μmol/L; AOBIOUS, Gloucester, MA, USA), osteocrin (Ocn, 100 nmol/l, R&D Systems, Minneapolis, Minnesota, USA) and cANP_4-23_ (100 nmol/l). Both cANP_4-23_ and Ocn are NPR-C ligands. While cANP_4-23_ acts as specific agonist of NPR-C, Ocn competes with NP for binding to NPR-C and blocks NPR-C dependent signalling pathways.^21,22^ To ensure functional neprilysin inhibition, the active agent sacubitrilat (formed from prodrug sacubitril by enzymatic cleavage of its ethyl ester group) was used. As previously described, the concentration of sacubitrilat was chosen to correspond to the concentration observed in blood plasma in a pharmacokinetic study designed to elucidate the respective plasma concentrations in the PARADIGM-HF trial, with the subjects receiving 200 mg of sacubitril/valsartan twice daily.^16,23,24^ Ca^2+^ spark measurements were conducted using a laser scanning confocal microscope (Leica TCS SP5, Leica, Wetzlar, Germany) with a 40× oil immersion objective. Fluorescence was excited by an argon ion laser (488 nm), and the emitted fluorescence was collected through a 505 nm long-pass emission filter. Fluorescence images were recorded in line-scan mode with 1400 lines per second, 512 pixels per line (with a width of 64 μm for each scan line). The experiments were conducted under resting conditions following the loading of the sarcoplasmic reticulum (SR) with Ca^2+^ through repetitive field stimulation for 10 s at 3 Hz. To minimize potential allocation bias and time-dependent experimental artefacts, all experimental groups were assigned to the different recording positions according to a predefined balanced rotation scheme. In addition, the maximum recording time per chamber was uniformly limited to 30 min to ensure consistent cell quality throughout all experiments. The Ca^2+^-sparks were analysed with the programme SparkMaster 2 (University of California Davis, Davis, California, USA,^25^) The Ca^2+^ spark frequency of the respective cell (CaSpF) was calculated by adjusting the number of sparks to cell width and scan rate and indicated as sparks per 100 µm per second (100 µm^−1^s^−1^).

### Epifluorescence microscopy

The isolated mouse cardiomyocytes were plated on laminin-coated chambers as described above. Subsequently, the cardiomyocytes were incubated with ratiometric fluorescent dye Indo-1 a.m. (10 µmol/L, Thermo Fisher Scientific, Waltham, MA, USA) for 15 min at room temperature, followed by a 10-min incubation with drug-free Tyrode’s solution for de-esterification at 37°C. Prior to the measurement, the solution was changed to either drug-free Tyrode’s in the control group or to a corresponding drug solution in the treatment groups and incubated for 5 min at 37°C. ATX-II (0.5 nmol/l) was used either as a standalone agent or in conjunction with one or more of the following active substances: CNP (100 nmol/l), sacubitrilat (40 µmol/l), and Ocn (100 nmol/l). During the experiments, a continuous superfusion with the corresponding drug solution was maintained. The measurements were conducted using a modified Ion-Optix fluorescence detection system (IonOptix LLC, Westwood, MA, USA).

The cells were excited at 340 nm and the emitted light was measured at 405 and 485 nm. The intracellular Ca^2+^ concentration was calculated as a ratio of the measured fluorescence (405/485 nm). Systolic Ca^2+^ transients were recorded at steady-state conditions under constant field stimulation (1 Hz). To evaluate the SR- Ca^2+^ content, the amplitude of caffeine-induced Ca^2+^ transients was quantified. Again, to minimize potential allocation bias and time-dependent experimental artefacts, all experimental groups were assigned to the different recording positions according to a predefined balanced rotation scheme. Following the cessation of stimulation during steady-state conditions at 1 Hz, caffeine (10 mmol/L; Sigma Aldrich, St. Louis, Missouri, USA) was directly applied onto the cell, resulting in immediate and complete SR Ca^2+^ release. The recorded Ca^2+^ transients were analysed using the software IonWizard® (IonOptix LLC, Westwood, MA, USA).

### Measurement of intracellular cGMP

Isolated murine ventricular cardiomyocytes were pre-incubated with phosphodiesterase inhibitor 3-Isobutyl-1-methylxanthin (IBMX, 0.5 mmol/l, Thermo Fisher Scientific, Waltham, MA, USA) for 10 min at 37°C. Afterwards cells were either incubated with CNP (100 nmol/l) or Sacubitrilat (40 µmol/l) for 10 min. Intracellular cGMP was extracted using ice-cold 70% ethanol and measured through radioimmunoassay as described before.^26,27^

### Generation of human induced pluripotent stem-cell-derived ventricular cardiomyocytes

Undifferentiated iPSCs, generated from a healthy male donor^21^ were maintained as a monolayer on Geltrex-coated (167 mg/ml; Thermo Fisher Scientific, Waltham, MA, USA) culture dishes in defined Essential 8 culture medium (Thermo Fisher Scientific, Waltham, MA, USA) and passaged twice a week using Versene (Thermo Fisher Scientific, Waltham, MA, USA). Written consent was obtained from the donors, and all investigations were performed in accordance with the principles outlined in the Declaration of Helsinki. Human iPSC-CM experiments were performed in compliance with the ethical committee of the University Medical Center Goettingen (Az -10/9/15) and Würzburg (2026–210z). For directed differentiation into ventricular cardiomyocytes, manipulation of the Wnt signalling pathway was employed, as previously described.^28–30^ Briefly, upon reaching 80–85% confluency, the medium was replaced with Cardio Differentiation medium (RPMI 1640 (Thermo Fisher Scientific, Waltham, MA, USA) with 0.05% albumin (Sigma Aldrich, St. Louis, MO, USA) and 0.02% L-Ascorbic Acid 2-Phosphate (Sigma Aldrich, St. Louis, MO, USA) supplemented with the GSK3-inhibitor CHIR99021 at 4 µmol/L (Millipore, Burlington, MA, USA) (day 0). After 48 h, the medium was changed to Cardio Differentiation medium with 5 µmol/L of IWP2 (Millipore, Burlington, MA, USA), an inhibitor of Wnt signalling. After 48 h, the medium was exchanged for Cardio Differentiation medium without supplementation for another 2 d. Afterwards, medium was refreshed with Cardio Culture medium (RPMI-1640 GlutaMax (Thermo Fisher Scientific, Waltham, MA, USA) with B27 supplement with insulin (Thermo Fisher Scientific, Waltham, MA, USA) every other day. The iPSC-CM were purified through metabolic selection using 4 mmol/L lactate in glucose-free RPMI medium (Thermo Fisher Scientific, Waltham, MA, USA) typically at 15–18 d post differentiation initiation.^31^ The purity of the ventricular iPSC-CMs was determined by flow analysis, with a focus on cardiac troponin T positivity (>85% cardiac TNT+), as well as through semiquantitative PCR analysis of ventricular-specific markers (MLC2v).

For semiquantitative PCR, total RNA was isolated using the SV Total RNA Isolation System (Promega) following the manufacturer’s protocol for RNA isolation from fibrous tissue. The DNase digestion step was extended to 30 min at room temperature. First-strand cDNA synthesis was performed using 100–300 ng of RNA with the iScript cDNA Synthesis Kit (Bio-Rad). For the PCR the following mix was prepared: 10 ng cDNA, 10 µM forward primer, 10 µM reverse primer, 2.5 mM d-NTPs (Invitrogen), 1× DREAM Taq buffer (Thermo Fisher), 0.625 U Dream Taq enzyme (Thermo Fisher) were combined with water to a total volume of 25 µl and run for 32 cycles with 30 s 56°C for the annealing and 30 s 72°C for the elongation steps. PCR products were subsequently run on a 1.5% agarose gel (110 V) supplemented with 5 µl Midori green (Nippon Genetics) Transilluminator (Herolab). Used primers are listed in Supplementary  *Table S1*. For flow analysis cells were fixed in 4% Histofix (Roth) for 20 min at room temperature. Afterwards cells were washed once in 1xPBS and subsequently stored in Blocking buffer (1% BSA/PBS) at 4°C until usage. For cTNT flow, cells were centrifuged at 300 g for 3 min at room temperature, and the pellet was washed once with 1 ml Staining buffer (0.1% Triton-X/1% BSA in 1× PBS). After a second centrifugation step, samples were resuspended in 200 µl antibody dilution (1:200 in Staining buffer) and incubated for 10–15 min at room temperature in the dark. Following incubation, cells were washed once with 1xPBS and resuspended in 500 µl 1xPBS for analysis using the MACSQuant16 (Miltenyi). For immunofluorescence, cells were incubated with primary antibody dilution (1:500 in Staining buffer) at 4°C. After 24 h, cells were washed three times with 1xPBS and incubated with secondary antibody dilution (1:500 in Staining buffer) for 1 h at room temperature. Subsequently cells were washed three times in Blocking buffer and once in water before being mounted onto microscope slides with Fluoromount-G (Southern Biotech) and imaged with Thunder-DMi8 widefield microscope (Leica). Used antibodies are listed in Supplementary  *Table S2*.

4–5 differentiation experiments were performed to generate ventricular iPSC-CMs. One week before Ca^2+^ measurements, 1–1.5 × 10^5^ of 60–90-day-old iPSC-CM were plated onto 35 mm glass bottom dishes. Cells were kept in the incubator at 37°C without stimulation (control) or with arrhythmic stimulation at 2 Hz with 40% cycle-length variability for 24 h before the measurement. Incubation with Fluo-4 and solution changes to either drug-free normal Tyrode’s solution (NT) or NT containing sacubitrilat (40 µmol/l) ± Ocn (100 nmol/l) were performed as described above. NT for iPSC contained (mmol/l) NaCl 140, KCL 5,4, MgCl_2_ 0.47, HEPES 10, glucose 10, CaCl_2_ 1,8 (pH 7,4, 37°C). Ca^2+^ spark measurements were conducted using a laser scanning confocal microscope (Eclipse Ti2, Nikon, Minato, Japan) with a 60× oil immersion objective. Fluorescence was excited by an argon ion laser (488 nm) and the emitted fluorescence was collected through a 505 nm long-pass emission filter. Fluorescence images were recorded in line-scan mode for 7,28 s, 1124 lines per second and 512 pixels per line (with a width of 44 μm for each scan line). The experiments were conducted under resting conditions following the loading of the SR with Ca^2+^ through repetitive field stimulation for 10 seconds at 2 Hz. CaSpF analysis was performed as described above.

### In silico modelling of potential binding sites of sacubitrilat in NPR-C

In silico prediction of sacubitrilat binding to NPR-C was performed by homology modelling and docking using the structures of NPR-C bound to CNP (PDB entry 1JDP,^32^) neprilysin in complex with sacubitrilat (PDB entry 5JMY^33^) and neprilysin bound to CNP (PDB entry 6SH2.^34^) It was hypothesized that sacubitrilat, in addition to inhibiting neprilysin, might also compete with CNP for binding to the extracellular domain of NPR-C. To follow this possibility and identify the mode of binding of sacubitrilat to NPR-C, a structural comparison of sacubitrilat and CNP was made. Based on the known binding site for CNP on NPR-C, regions involved in hydrophobic interactions were prioritized. Given the prominent biphenyl group of sacubitrilat, which mediates key hydrophobic contacts in neprilysin, sacubitrilat was manually positioned at the corresponding hydrophobic pocket in NPR-C occupied by the Met residue 17 of CNP. A short *in vacuo* molecular dynamics simulation (YASARA, NOVA force field,^35^) was subsequently performed to relax the manually docked sacubitrilat.

### Statistics

Data were analysed using GraphPad Prism 10 (GraphPad Software, Boston, MA, USA). All data are presented as mean ± standard error of the mean. One-way ANOVA with Tukey's *post hoc* test was used for multiple comparisons. *P* values < .05 were considered statistically significant.

## Results

### Effects of natriuretic peptides on SR-Ca^2+^ leak in murine wild-type cardiomyocytes

Ca^2+^ sparks are spontaneous Ca^2+^ release events from the SR that are proarrhythmic. Through elimination of the released Ca^2+^ via the electrogenic NCX, a dissipation of the membrane potential can be induced sufficient to induce a delayed afterdepolarization, which—if propagating throughout the ventricle on the whole heart level-can induce premature ventricular contractions or even ventricular fibrillation. Therefore, we determined the frequency of Ca^2+^ sparks (CaSpF) in murine ventricular cardiac myocytes as a readout that can predict the pro- or anti-arrhythmic effects of different interventions.

Compared with control conditions, the addition of ATX-II increased CaSpF two-fold (*Figure 1A and B*). This effect was abolished after administration of CNP, which lowered CaSpF compared with ATX-II by 38 ± 5% (*Figure 1A and B*). In contrast, ANP did not significantly lower CaSpF compared with ATX-II (*Figure 1A and B*). The addition of CNP, but not ANP to ATX-II significantly increased CaSp-amplitude compared with ATX-II alone (*Figure 1C*).

**Figure 1:**
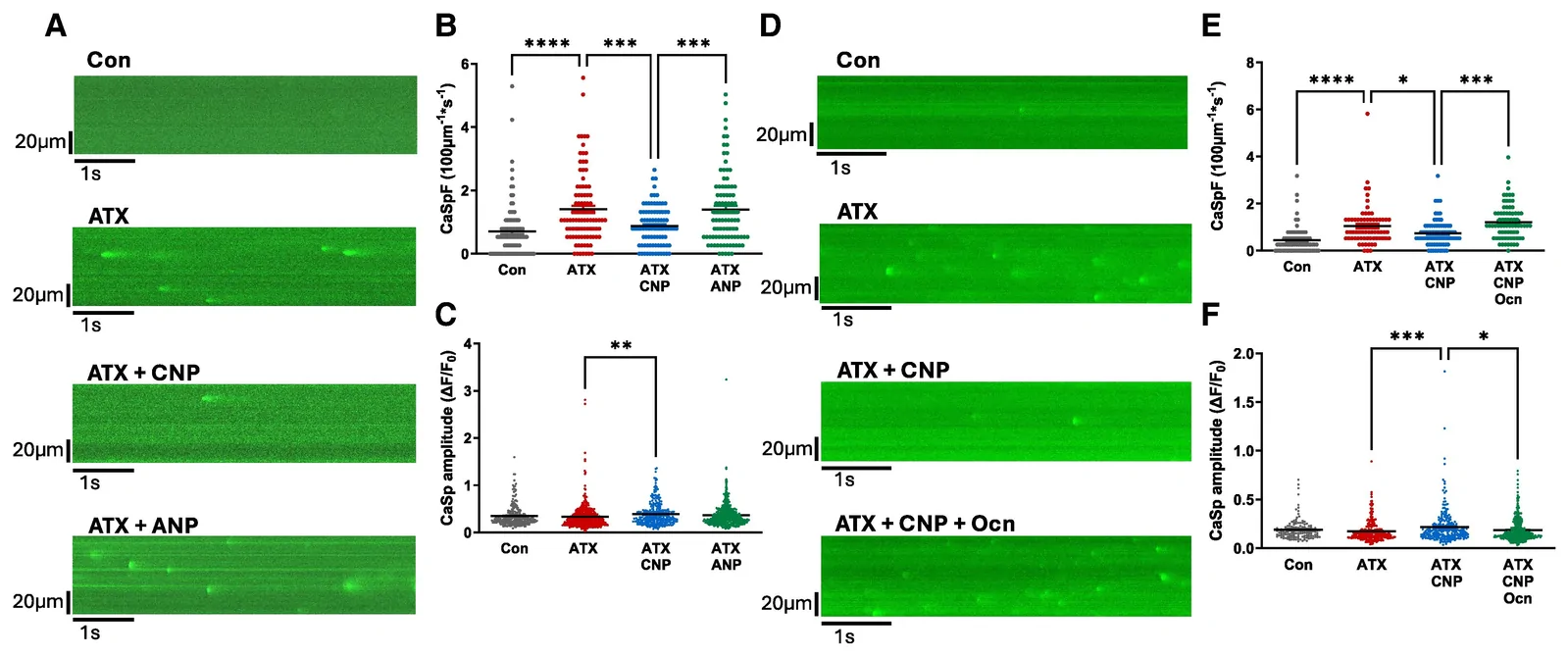
Effects of ANP and CNP ± Ocn on ATX-II-induced SR Ca^2+^ leak in murine wild-type cardiomyocytes. (*A*) and (*D*) Representative confocal line scans of murine ventricular cardiomyocytes; (*B*) CaSpF was significantly increased by ATX-II compared with control (Con vs ATX-II: 0.7 ± 0.09 vs 1.4 ± 0.12 × 100 µm^−1^s^−1^; n cells/hearts = 95/6 each, *P* < .001), CNP significantly lowered CaSpF compared with ATX-II (ATX-II vs ATX-II + CNP: 1.4 ± 0.12 vs 0.87 ± 0.07 × 100 µm^−1^s^−1^; n cells/hearts = 95/6 vs 87/6, *P* < .001), ANP had no effect on CaSpF (ATX-II vs ATX-II + ANP: 1.4 ± 0.12 vs 1.4 ± 0.12 × 100 µm^−1^s^−1^, n cells/hearts = 95/6 each, *P* = .99). (*C*) CNP significantly elevated CaSp-amplitude compared with ATX-II (ATX-II vs ATX-II + CNP: ΔF/F_0_: 0.33 ± 0.11 vs 0.39 ± 0.011, n cells/hearts = 95/6 vs 87/6, *P* = .003), in contrast to ANP (ATX-II vs ATX-II + ANP: ΔF/F_0_: 0.33 ± 0.11 vs 0.37 ± 0.01, n cells/hearts = 95/6 vs 95/6, *P* = .098). (*E*) Ocn blunted CNP-induced effects on CaSpF (ATX-II + CNP vs ATX-II + CNP + Ocn: 0.75 ± 0.06 vs 1.22 ± 0.085 × 100 µm^−1^s^−1^, n cells/hearts = 78/5 vs 74/5, *P* < .001; ATX-II vs ATX-II + CNP + Ocn: 1.05 ± 0.07 vs 1.22 ± 0.085 × 100 µm^−1^s^−1^, n cells/hearts = 70/5 vs 74/5, *P* = .52). (*F*) CNP significantly elevated CaSp-amplitude compared with ATX-II (ATX-II vs ATX-II + CNP: ΔF/F_0_: 0.172 ± 0.006 vs 0.22 ± 0.011, n cells/hearts = 70/5 vs 78/5, *P* < .001). This effect was blunted after co-incubation with Ocn (ATX-II vs ATX-II + CNP + Ocn: ΔF/F_0_: 0.172 ± 0.006 vs 0.185 ± 0.006, n cells/hearts = 70/5 vs 74/5, *P* = .57). A one-way ANOVA with a Tukey *post hoc* test was used for the statistical analysis. * = *P* < .05; ** = *P* < .01; *** = *P* < .001; **** = *P* < .0001

To further evaluate which CNP receptor (GC-B vs NPR-C) is responsible for the reduction of the diastolic Ca^2+^-leak, in a second set of experiments, we again increased CaSpF with ATX-II, prevented this increase with CNP, and then blocked NPR-C with Ocn (*Figure 1D and E*). Notably, Ocn abolished the protective effect of CNP on the diastolic Ca^2+^-leak. Moreover, the CNP-induced increase in CaSp-amplitude compared with ATX-II alone was also blunted by co-incubation with the NPR-C blocker Ocn (*Figure 1F*). To exclude unspecific effects of Ocn, we performed CaSp measurements with sole Ocn treatment. Ocn neither affected CaSpF under basal conditions nor in response to ATX-II (*Figure 2A, and B*). In contrast, specific activation of NPR-C with cANP_4-23_ blunted the ATX-II-induced increase in CaSpF, thereby mimicking the effect of CNP (*Figure 2C and D*). Together, these data indicate that activation of the late INa increases spontaneous Ca^2+^ release events from the SR, is blunted by activation of NPR-C with CNP or cANP_4-23_, while ANP has no effects.

**Figure 2:**
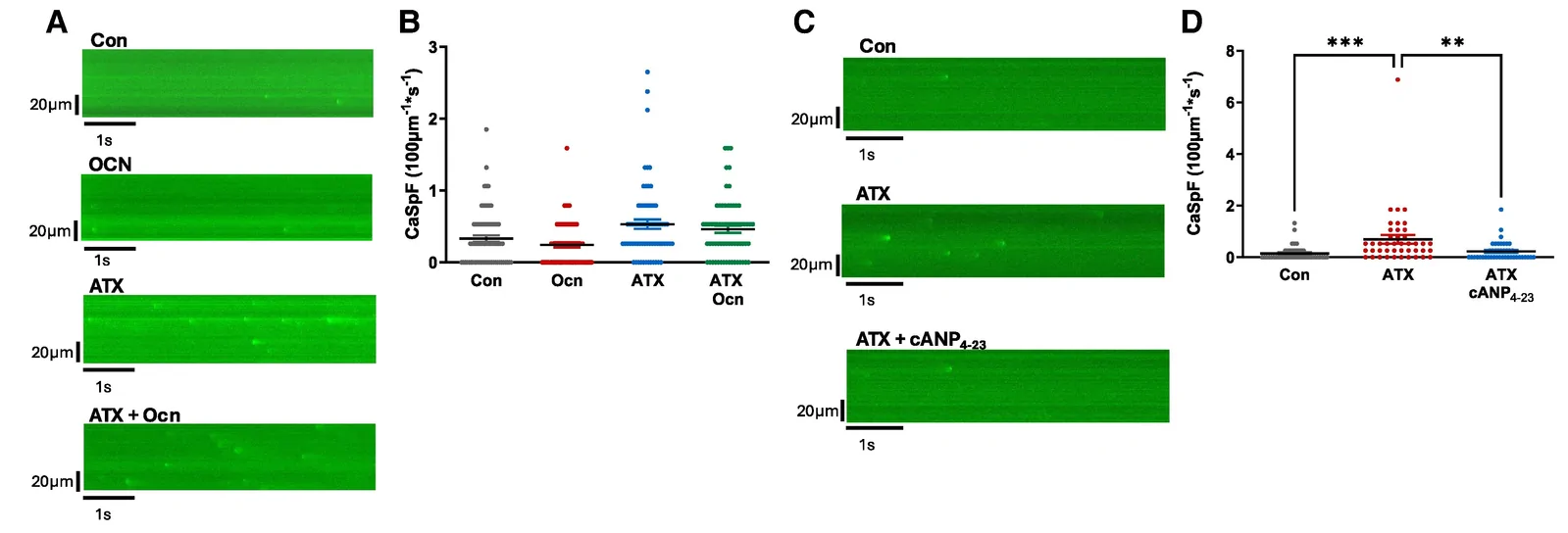
Effects of Ocn under basal conditions and on ATX-induced diastolic SR Ca^2+^ leak as well as cANP_4-23_ on ATX-induced SR Ca^2+^ leak in murine wild-type cardiomyocytes. (*A*) and (*C*) Representative confocal line scans of murine ventricular cardiomyocytes; (*B*) Ocn did not significantly change CaSpF under basal conditions (Con vs Ocn: 0.33 ± 0.04 vs 0.24 ± 0.04 × 100 µm^−1^s^−1^, n cells/hearts = 70/4 vs 69/4, *P* = .51) as well as under stimulation with ATX-II (ATX-II vs ATX-II + Ocn: 0.53 ± 0.06 vs 0.46 ± 0.05 × 100 µm^−1^s^−1^, n cells/hearts = 68/4 vs 67/4, *P* = .68). (*D*) cANP_4-23_ significantly reduced CaSpF compared with ATX-II (0.69 ± 0.12 vs 0.23 ± 0.05 × 100 µm^−1^s^−1^, n cells/hearts = 43/3 each, *P* = .006). A one-way ANOVA with a Tukey *post hoc* test was used for the statistical analysis. ** = *P* < .01; *** = *P* < .001

### Effects of sacubitrilat on SR-Ca^2+^ leak in murine wild-type cardiomyocytes

To determine whether and how sacubitrilat can prevent ATX-II-induced Ca sparks, we co-incubated ATX-II with sacubitrilat alone or a combination of sacubitrilat with the NPR-C blocker Ocn. Sacubitrilat completely blunted the ATX-II-induced increase in CaSpF, and this protection was completely reversed by blocking NPR-C with Ocn (*Figure 3A and B*). Co-incubation of ATX-II with sacubitrilat increased the CaSp-amplitude compared with ATX-II alone, and this effect of sacubitrilat was prevented by Ocn (*Figure 3C*).

**Figure 3:**
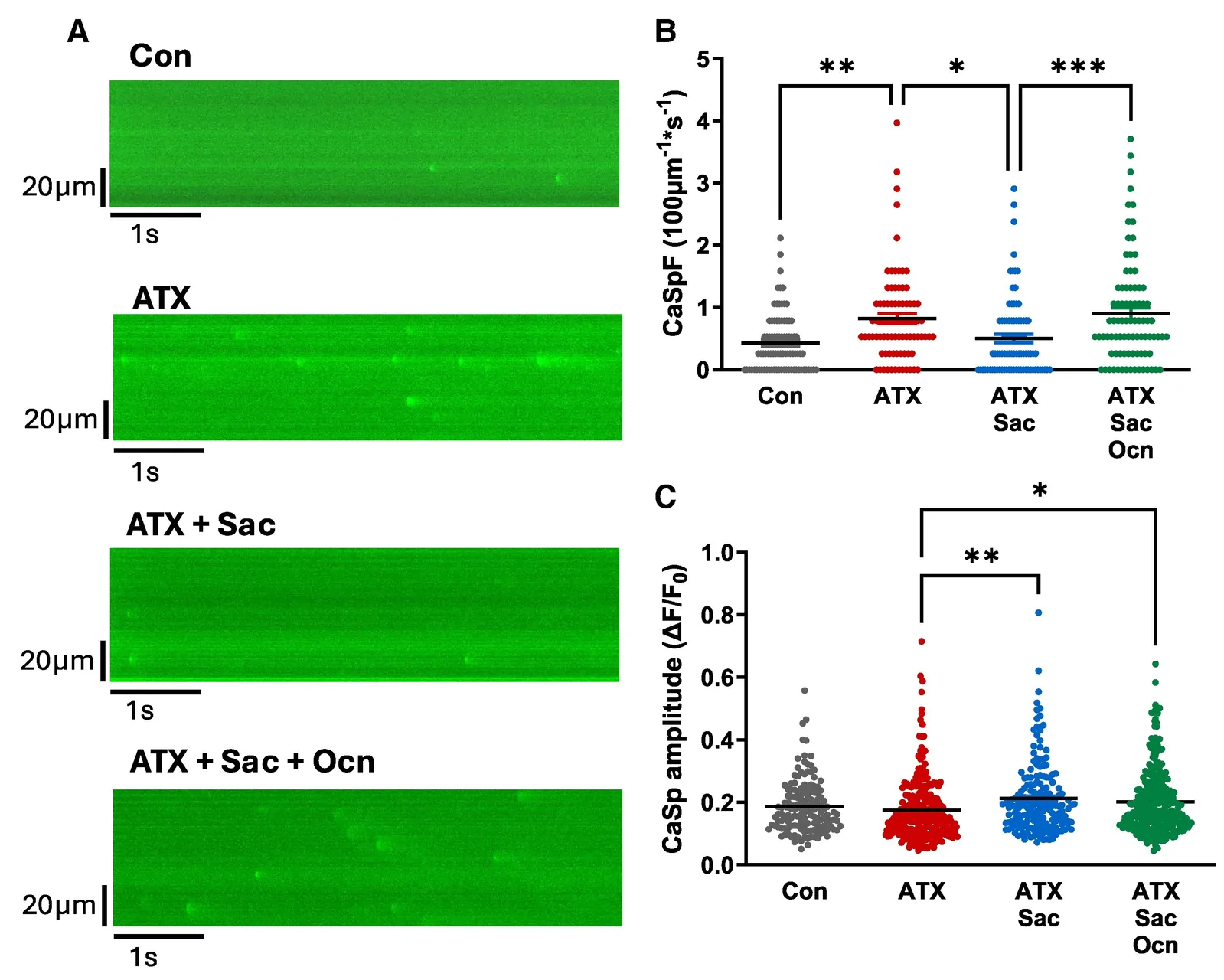
Effects of Sac ± Ocn on ATX- induced SR Ca^2+^ leak in murine ventricular cardiomyocytes. (*A*) Representative confocal line scans of murine ventricular cardiomyocytes; (*B*) Sac significantly reduced CaSpF compared with ATX-II (ATX-II vs ATX-II + Sac: 0.82 ± 0.08 vs 0.51 ± 0.07 × 100 µm^−1^s^−1^, n cells/hearts = 84/5 vs 87/5, *P* = .014). Upon Co-Incubation with Ocn the effect on CaSpF was blunted (ATX-II vs ATX-II + Sac + Ocn: 0.82 ± 0.08 vs 0.9 ± 0.09 × 100 µm^−1^s^−1^, n cells/hearts = 84/5 vs 86/5, *P* = .87; ATX-II + Sac vs ATX-II + Sac + Ocn: 0.51 ± 0.07 vs 0.9 ± 0.09 × 100 µm^−1^s^−1^, n cells/hearts = 87/5 vs 86/5, *P* = .001). (*C*) Sac increased CaSp-amplitude compared with ATX-II (ATX-II vs ATX-II + Sac: ΔF/F_0_: 0.175 ± 0.006 vs 0.21 ± 0.009, n cells/hearts = 84/5 vs 87/5, *P* = .001), but not upon co-incubation with Ocn (ATX-II vs ATX-II + Sac + Ocn: ΔF/F_0_: 0.175 ± 0.006 vs 0.2 ± 0.006, n cells/hearts = 84/5 vs 86/5, *P* = .69). A one-way ANOVA with a Tukey *post hoc* test was used for the statistical analysis. * = *P* < .05; ** = *P* < .01; *** = *P* < .001

### Effects of CNP and sacubitrilat on systolic Ca^2+^ release and Ca^2+^ reuptake in murine ventricular cardiomyocytes

As the arrhythmogenic SR Ca^2+^ leak is not only determined by the closure properties of the RyR2-clusters but also by the SR Ca^2+^ load (as driving force), levels of SR Ca^2+^-storage as well as systolic Ca^2+^ release and Ca^2+^ reuptake dynamics were evaluated under steady-state conditions during 1 Hz pacing in all treatment groups. After ATX-II incubation, the amplitude of the systolic Ca^2+^ transients (ΔF_405_/F_485_) was modestly increased compared with control (*Figure 4A and B*). CNP co-stimulation with ATX-II did not significantly affect steady-state systolic Ca^2+^ transient amplitude compared with ATX-II alone, nor did the combined incubation of ATX-II with CNP and the NPR-C inhibitor Ocn (*Figure 4A and B*). Diastolic Ca^2+^ concentrations were not affected by any of these interventions (*Figure 4A and C*). Despite unchanged diastolic Ca^2+^, the rate of cytosolic Ca^2+^ decay, which in murine cardiac myocyte is primarily driven by SR Ca^2+^ ATPase (SERCA) activity, was accelerated by CNP when combined with ATX-II compared with ATX-II alone, while additional co-incubation with Ocn did not blunt this CNP-mediated effect (*Figure 4A and D*). Also, when adding sacubitrilat to ATX-II, no increase in systolic or diastolic Ca^2+^ concentrations or acceleration of Ca^2+^ decay kinetics were observed, but when adding Ocn to sacubitrilat and ATX-II, both systolic and diastolic Ca^2+^ concentrations increased and diastolic Ca^2+^ kinetics accelerated (*Figure 4E–H*). Together, these data indicate that neither CNP nor sacubitrilat affect steady-state Ca^2+^ concentrations.

**Figure 4:**
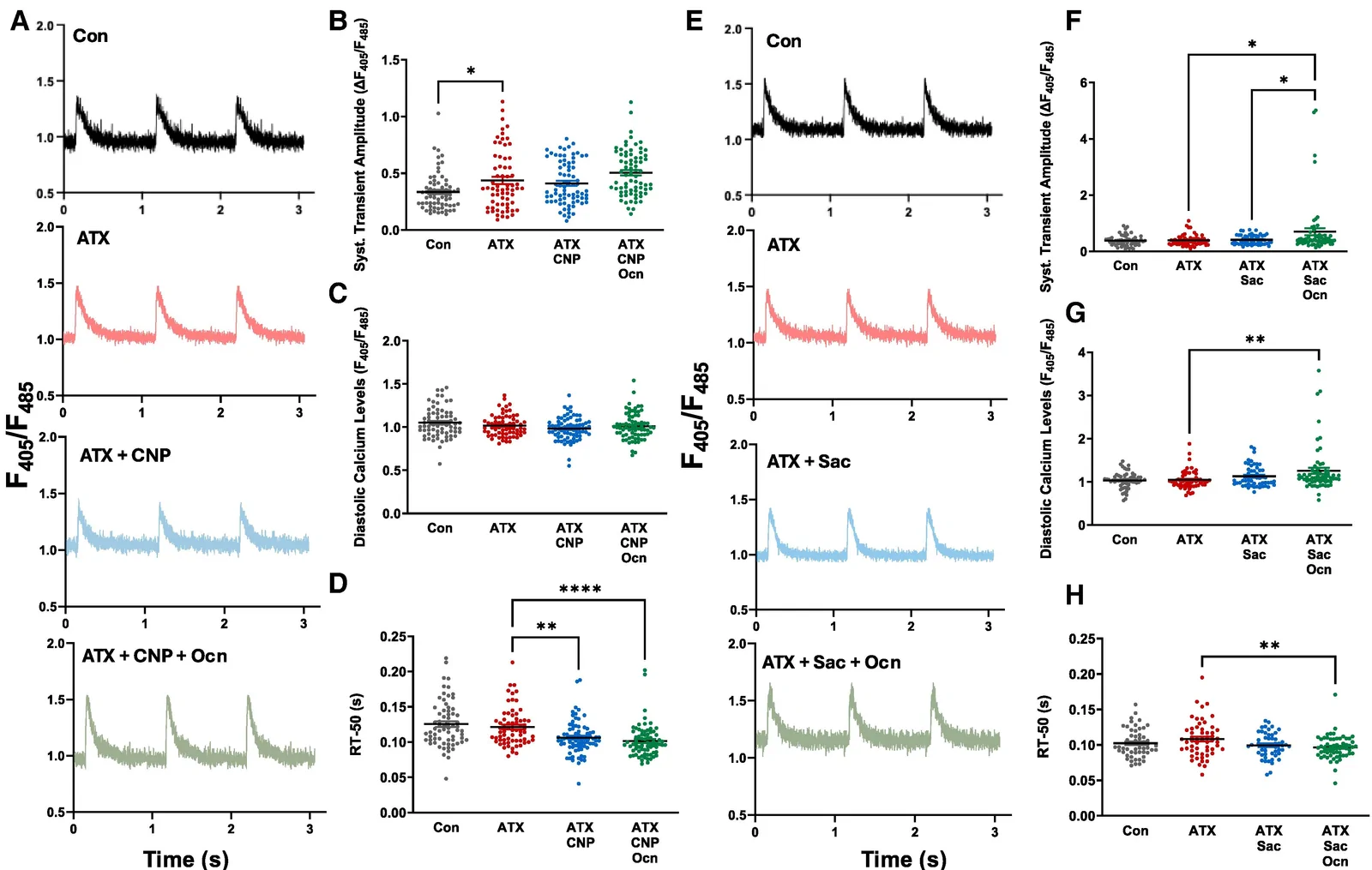
*Effects of CNP and Sac* ± Ocn on calcium transients and kinetics. (*A–D*) CNP as well as CNP ± Ocn did not significantly change calcium transient amplitude (ATX-II vs ATX-II + CNP: ΔF_405_/F_485_: 0.43 ± 0.03 vs 0.41 ± 0.02, n cells/hearts = 66/5 vs 76/5, *P* = .89; ATX-II vs ATX-II + CNP + Ocn: ΔF_405_/F_485_: 0.43 ± 0.03 vs 0.50 ± 0.02, n cells/hearts = 66/5 vs 73/5, *P* = .23) as well as diastolic calcium load compared with ATX-II (ATX-II vs ATX-II + CNP: F_405_/F_485_: 1.02 ± 0.015 vs 0.98 ± 0.015, n cells/hearts = 68/5 vs 76/5, *P* = .46.; ATX-II vs ATX-II + CNP + Ocn: F_405_/F_485_: 1.02 ± 0.015 vs 1.01 ± 0.018, n cells/hearts = 68/5 vs 73/5, *P* = .99). RT-50 was significantly reduced by CNP and CNP + Ocn compared with ATX-II (ATX-II vs ATX-II + CNP: 0.122 ± 0.003 vs 0.106 ± 0.003 s, n cells/hearts = 66/5 vs 76/5, *P* = .003; ATX-II vs ATX-II + CNP + Ocn: 0.122 ± 0.003 vs 0.101 ± 0.003 s, n cells/hearts = 66/5 vs 73/5, *P* < .001). (*E–H*) Sac did not influence calcium transient amplitude (ATX-II vs ATX-II + Sac: 0.39 ± 0.03 vs 0.41 ± 0.02, n cells/hearts = 56/4 vs 52/4, *P* = .97), diastolic calcium load (ATX-II vs ATX-II + Sac: 1.04 ± 0.03 vs 1–13 ± 0.03, n cells/hearts = 56/4 vs 54/4, *P* = .54) and RT-50 (ATX-II vs ATX-II + Sac: 0.108 ± 0.003 vs 0.099 ± 0.002, n cells/hearts = 56/4 vs 52/4, *P* = .11) compared with ATX-II. Co-incubation of Sac and osteocrin lead to significantly higher calcium transient amplitudes (ATX-II vs ATX-II + Sac + Ocn: 0.39 ± 0.03 vs 0.7 ± 0.12, n cells/hearts = 56/4 vs 60/4, *P* = .01) and higher diastolic calcium load (ATX-II vs ATX-II + Sac + Ocn: 1.04 ± 0.03 vs 1.25 ± 0.07, n cells/hearts = 56/4 vs 62/4, *P* = .004) and to a significantly lower RT-50 (ATX-II vs ATX-II + Sac + Ocn: 0.108 ± 0.003 vs 0.096 ± 0.002 s, n cells/hearts = 56/4 vs 60/4, *P* = .01) compared with ATX-II. A one-way ANOVA with a Tukey *post hoc* test was used for the statistical analysis. * = *P* < .05; ** = *P* < .01; **** = *P* < .0001

The half-maximum relaxation time (RT-50) reflecting the activities of SERCA and NCX remained unchanged upon stimulation with ATX-II compared with control (*Figure 4A and D*). CNP and CNP + Ocn significantly reduced RT-50 compared with ATX-II (*Figure 4A and D*) hinting towards a GC-B dependent effect activating SERCA activity. Sacubitrilat did not influence RT-50 compared with ATX-II (*Figure 4E and H*). Sacubitrilat + Ocn significantly lowered RT-50 compared with ATX-II (*Figure 4E and H*).

### Effects of CNP and sacubitrilat on SR Ca^2+^ load

To estimate SR Ca^2+^ load, we measured caffeine-induced Ca^2+^ transients (*Figure 5*). SR Ca^2+^ load remained unchanged after stimulation with ATX-II (*Figure 5A and B*). Upon stimulation with CNP as well as with CNP + Ocn, caffeine-induced transients did not change significantly compared with sole ATX-II treatment (*Figure 5A and B*). Sacubitrilat did not significantly modify SR Ca^2+^ load compared with ATX-II (*Figure 5E and F*). Co-Incubation of sacubitrilat and Ocn, however, led to a significantly higher SR Ca^2+^ load compared with sole sacubitrilat treatment but not compared with ATX-II (*Figure 5E and F*).

**Figure 5:**
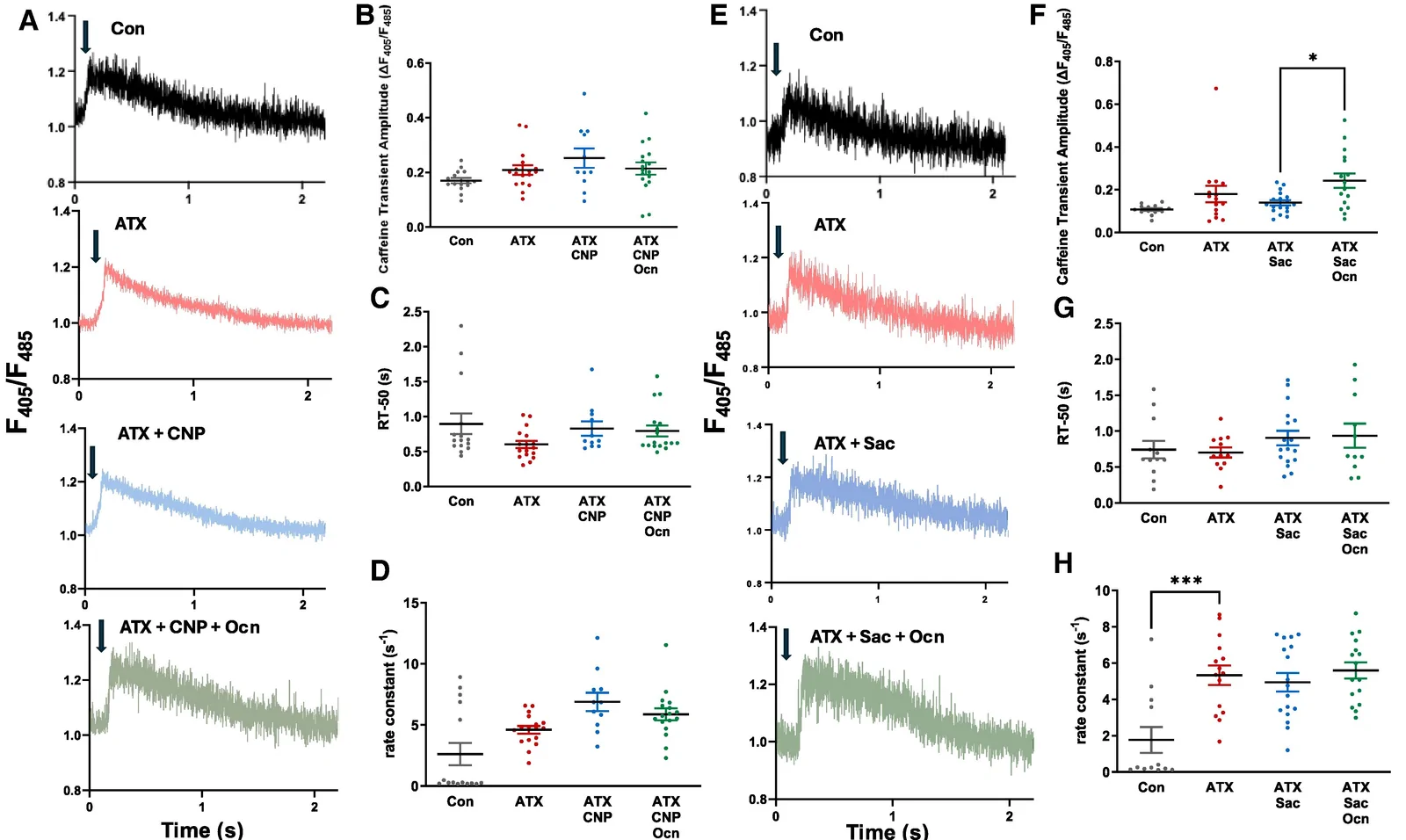
*Effects of CNP and Sac* ± Ocn on caffeine induced calcium transients. (*A–D*) CNP alone and in combination with Ocn did not significantly influence caffeine-induced Ca^2+^-transient amplitude (ATX-II vs ATX-II + CNP: ΔF_405_/F_485_: 0.21 ± 0.017 vs 0.25 ± 0.036, n cells/hearts = 17/5 vs 11/4, *P* = .08; ATX-II vs ATX-II + CNP + Ocn: ΔF_405_/F_485_: 0.21 ± 0.02 vs 0.21 ± 0.02, n cells/hearts = 17/5 vs 17/5, *P* = .43), RT-50 (ATX-II vs ATX-II + CNP: 0.6 ± 0.005 s vs 0.82 ± 0.1 s, n cells/hearts = 17/5 vs 11/4, *P* = .41; ATX-II vs ATX-II + CNP + Ocn: 0.6 ± 0.05 s vs 0.79 ± 0.08 s, n cells/hearts = 17/5 vs 17/5, *P* = .45) or the rate constant reflecting SERCA activity (ATX-II vs ATX-II + CNP: 4.6 ± 0.3 s^−1^ vs 6.9 ± 0.75 s^−1^, n cells/hearts = 17/5 vs 11/5, *P* = .08; ATX-II vs ATX-II + CNP + Ocn: 4.6 ± 0.3 s^−1^ vs 5.9 ± 0.05 s^−1^, n cells/hearts = 17/5 vs 17/5, *P* = .43). (*E–H*) Sac as well as Sac + Ocn did not significantly change caffeine-induced Ca^2+^-transient amplitude (ATX-II vs ATX-II + Sac: ΔF_405_/F_485_: 0.18 ± 0.04 vs 0.14 ± 0.012, n cells/hearts = 15/5 vs 18/5, *P* = .67;, ATX-II vs ATX-II + Sac + Ocn: ΔF_405_/F_485_: 0.18 ± 0.04 vs 0.24 ± 0.03, n cells/hearts = 15/5 vs 16/5, *P* = .36), RT-50 (ATX-II vs ATX-II + Sac: 0.7 ± 0.07 s vs 0.9 ± 0.1 s, n cells/hearts = 12/5 vs 17/5, *P* = .59; ATX-II vs ATX-II + Sac + Ocn: 0.7 ± 0.07 s vs 0.93 ± 0.17 s, n cells/hearts = 12/5 vs 11/5, *P* = .55) and the rate constant compared with ATX-II (ATX-II vs ATX-II + Sac: 4.9 ± 0.46 s^−1^ vs 4.9 ± 0.51 s^−1^, n cells/hearts = 15/5 vs 17/5, *P* = .95; ATX-II vs ATX-II + Sac + Ocn: 4.9 ± 0.46 s^−1^ vs 5.6 ± 0.44 s^−1^, n cells/hearts = 15/5 vs 16/5, *P* = .98). A one-way ANOVA with a Tukey *post hoc* test was used for the statistical analysis. * = *P* < .05; *** = *P* < .001

The RT-50 in caffeine-induced transients reflecting only NCX activity remained unchanged after incubation with CNP as well as with CNP + Ocn (*Figure 5A and C*). Sacubitrilat alone as well as in combination with Ocn did not change RT-50 in caffeine-induced Ca^2+^ transients compared with ATX-II (*Figure 5E and G*).

To approximate SERCA activity (k_SERCA_) as previously reported we subtracted the rate constant of decay (k) of the caffeine-triggered transient (k_caff_) from that of the systolic Ca^2+^ transient (k_SERCA_ = k_sys_—k_caff_).^36^ CNP as well as CNP + OCN showed a not significant trend towards a higher Serca activity (k_SERCA_) compared with ATX-II (*Figure 5D*). Sacubitrilat as well as Sacubitrilat + Ocn did not significantly change k_SERCA_ compared with ATX-II *(Figure 5H*).

### Effects of CNP and sacubitrilat on cGMP-levels

The impact of sacubitrilat on cardiomyocyte cGMP levels was determined by radioimmunoassay. As shown, CNP led to a 3.25-fold increase in cGMP levels (compared with vehicle/PBS treatment), while sacubitrilat did not increase cGMP levels (*Figure 6*).

**Figure 6:**
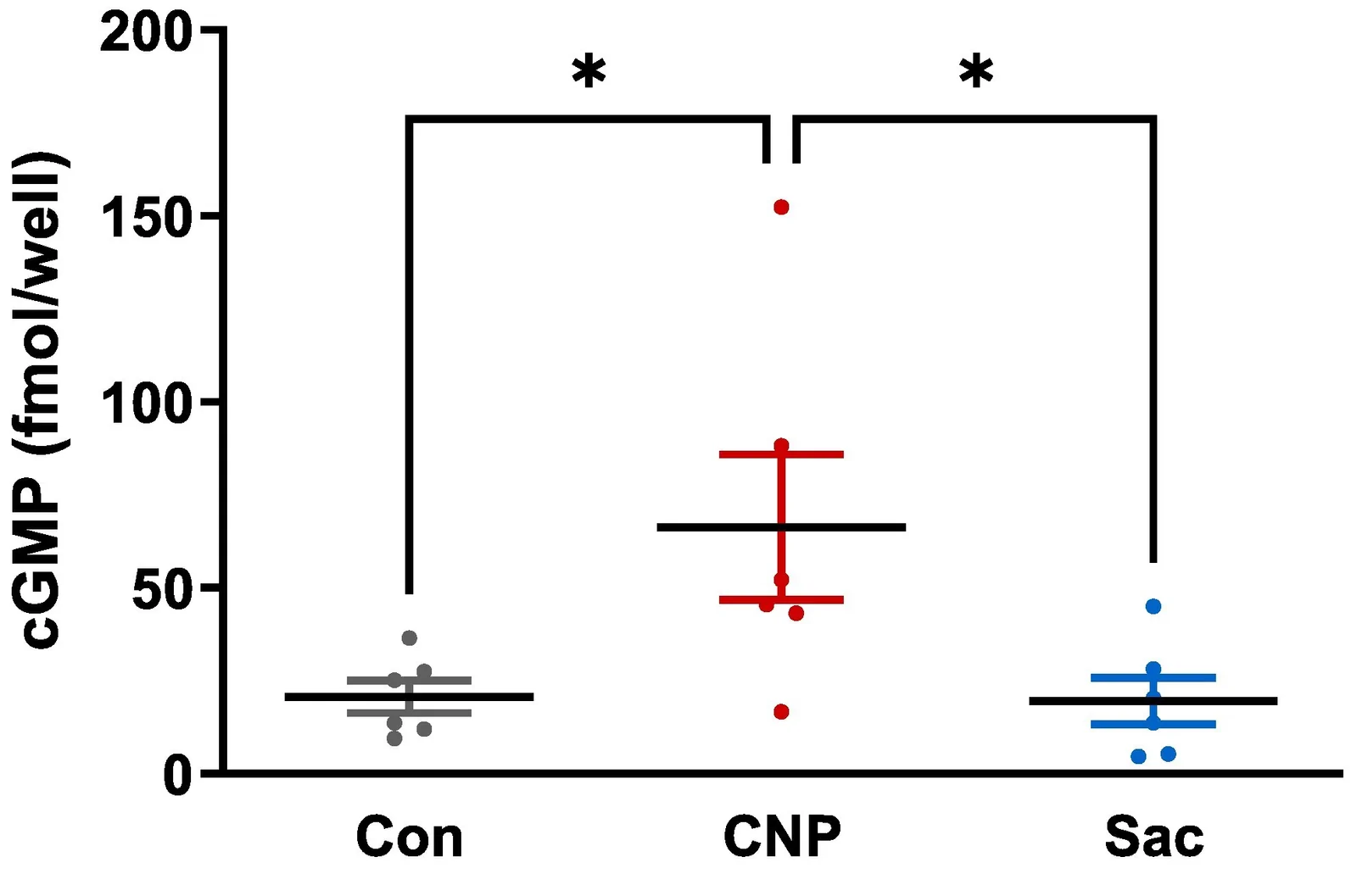
Impact of CNP and Sac on intracellular cGMP levels. CNP significantly elevated cGMP levels compared with control (Con vs CNP: 20.8 ± 4.35 vs 66.42 ± 19.58 fmol/well, n samples/mice: 6/3, *P* = .04), whereas Sac did not significantly influence cGMP levels (con vs sac: 20.8 ± 4.35 vs 19.6 ± 6.26 fmol/well, n samples/mice: 6/3, *P* = .99). A one-way ANOVA with a Tukey *post hoc* test was used for the statistical analysis. * = *P* < .05

### Effects of sacubitrilat on SR-Ca^2+^ leak in iPSC-CM

To ensure the transferability of our results to the treatment of human cardiac pathologies, we additionally performed experiments in human ventricular iPSC-CMs. Results of the quality control can be found in Supplementary  *Figure S1*. 24 h tachyarrhythmic stimulation (2 Hz with 40% cycle-length variability, *Figure 7B*) of iPSC-CM led to a significant increase in CaSpF compared with control cells (*Figure 7A and C*). Importantly, sacubitrilat also reduced CaSpF in this model of human Arrhythmia-induced cardiomyopathy, while co-incubation with Ocn blocked this effect.

**Figure 7:**
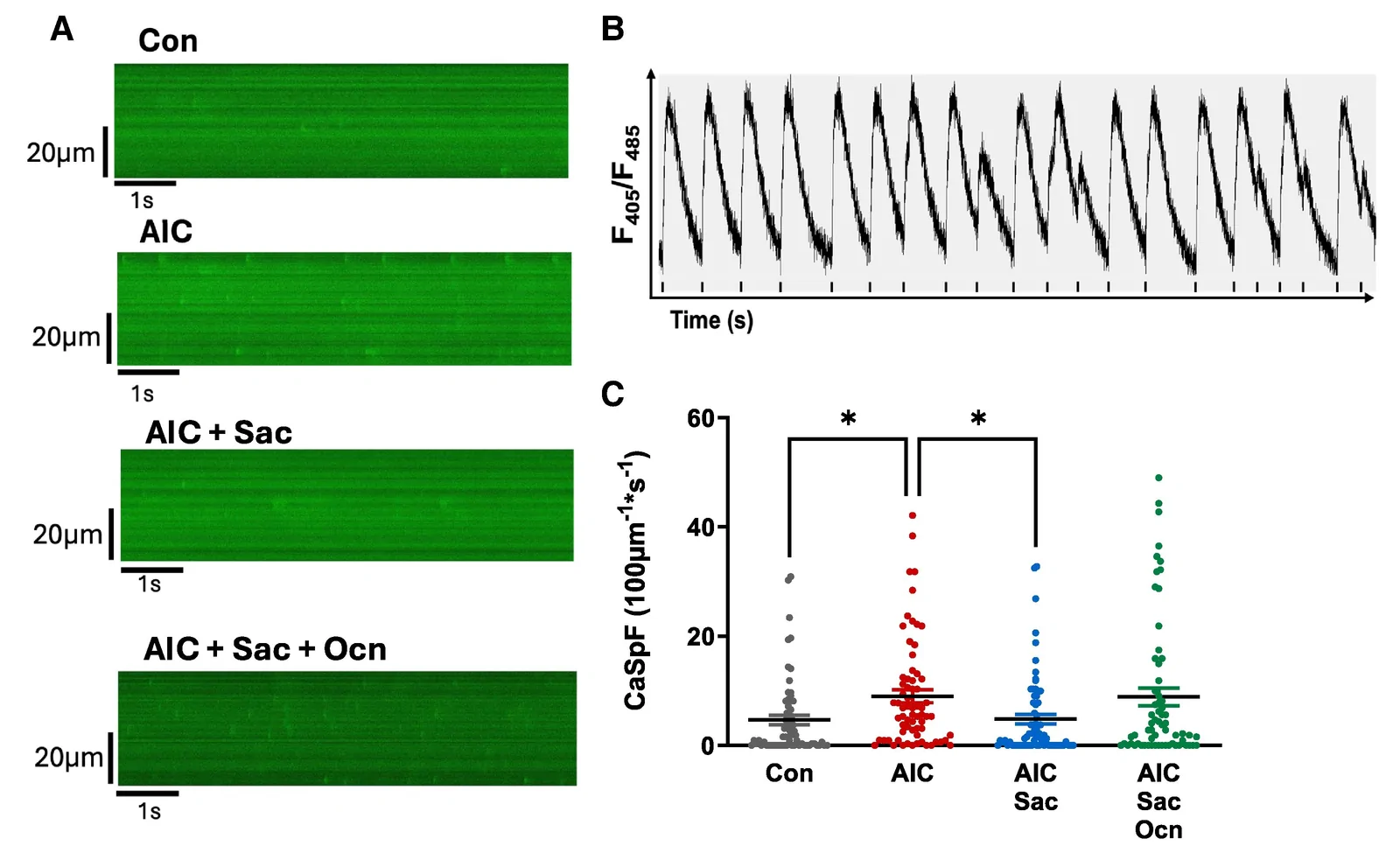
Sac reduces SR-Ca^2+^ leak in an AIC model in iPSC-CM dependent on NPR-C. (*A*) Original representative line scans of iPSC-CM; (*B*) Original calcium transient (Indo-1) of an iPSC-CM under tachyarrhythmic stimulation recorded by epifluorescence microscopy. (*C*) CaSpF is significantly elevated by 24-h tachyarrhythmic stimulation compared with control (Con vs AIC: 4.66 ± 0.85 vs 9.03 ± 1.18 × 100 µm^−1^s^−1^, n cells/differentiations = 67/4 vs 68/4, *P* = .04) and again reduced to control level by Sac treatment (AIC vs AIC + Sac: 9.03 ± 1.18 vs 4.80 ± 0.88 × 100 µm^−1^s^−1^, n cells/differentiations = 68/4 vs 70/4, *P* = .047). The effect is blunted upon co-incubation with Ocn (AIC vs AIC + Sac + Ocn: 9.03 ± 1.18 vs 8.94 ± 1.16 × 100 µm^−1^s^−1^, n cells/differentiations = 67/4 vs 68/4, *P* = .99). A one-way ANOVA with a Tukey *post hoc* test was used for the statistical analysis. * = *P* < .05

### In silico docking of sacubitrilat to NPR-C

A comparison of the published structures of neprilysin with sacubitrilat^30^ or CNP^29^ show that both sacubitrilat and CNP bind to neprilysin using an overlapping epitope and in a competing manner. This suggests that sacubitrilat and the CNP peptide share structural similarities and sacubitrilat can in part mimic the binding of CNP. Hence, we assumed that sacubitrilat might also directly bind to NPR-C and possibly competes with CNP. Unfortunately, the amino acid sequence of the stretch of CNP bound to neprilysin could not be identified in the crystal structure (PDB entry 1JDP) and was hence modelled as four alanine residues instead. To identify this sequence-stretch in CNP that mimics sacubitril binding to neprilysin, AlphaFold 3 structure predictions for neprilysin-CNP complexes were made. Three CNP fragments were tested in these AlphaFold structure predictions comprising either Gly1 to Gly8, residues Phe7 to Arg13, or residues Leu11 to Gly19 of CNP. These runs suggested that a large hydrophobic amino acid residue such as Phe7 or Met17 can mimic the biphenyl group of sacubitrilat.

Therefore, using the software Yasara sacubitrilat was manually docked into the CNP binding site of NPR-C such that the biphenyl group of sacubitrilat occupied the cleft filled with Met17 of CNP. Sacubitrilat was then oriented such that the carboxylate groups located near NPR-C Arg166 and Arg211 of the other protomer of the NRP-C dimer. To relax steric restraints from the manual placement, short energy minimization and *in vacuo* molecular dynamics simulations were performed. The biphenyl group of sacubitrilat occupies a hydrophobic pocket comprising Tyr140, Phe164 and Tyr171 of NPR-C reasonably well, consistent with the size and character of the native peptide interaction (*Figure 8*). Both carboxylate groups form extensive hydrogen bonding with the two arginine residues, i.e. Arg166 and Arg 211, the amide group of sacubitrilat engages in an H-bond interaction with the backbone carbonyl group of NPR-C Lys209. When comparing our model of sacubitrilat bound to NPR-C with the complex of sacubitrilat and neprilysin, similar interactions are found. The biphenyl group is surrounded by aromatic amino acid residues of neprilysin, one carboxylate group is also coordinated by two arginine residues of neprilysin, while the second carboxylate group of sacubitrilat is interacting with a zinc immobilized in neprilysin. Together, these data indicate that sacubitrilat may bind to the similar binding pocket in neprilysin as CNP does thereby possibly acting as competitive inhibitor of CNP.

**Figure 8:**
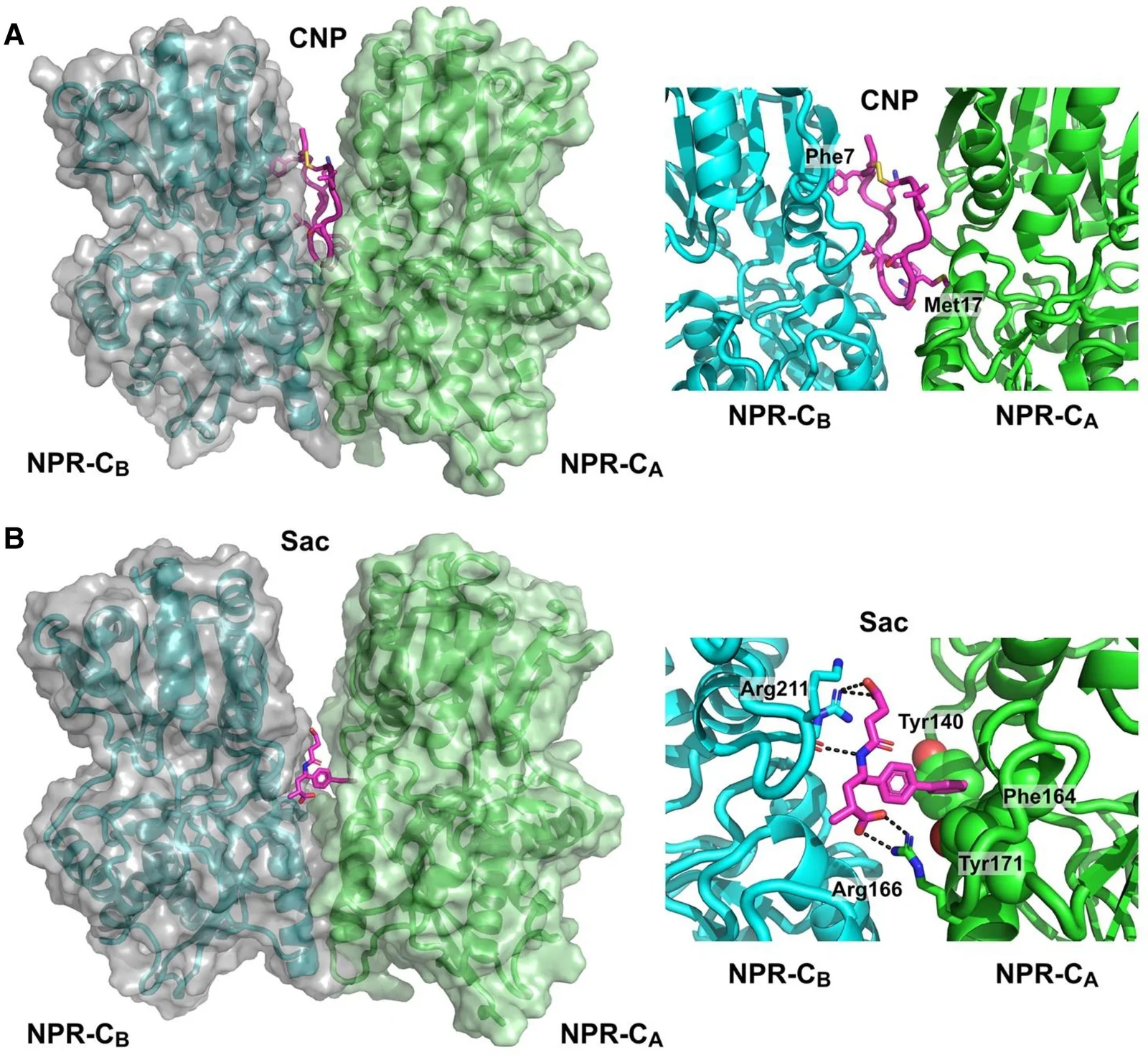
In silico docking of sacubitril to NPR-C. (*A*) Structure of CNP (marked in magenta) bound to two NPR-C (shown in green and cyan, protein surface is indicated in grey and light green). The cyclic CNP is bound between the NPR-C dimer interface with two large hydrophobic residues, i.e. Phe7 and Met17 protruding into hydrophobic pockets in the NPR-C protomers (magnified cut-out in the right panel). (*B*) Model of sacubitril (marked in magenta) bound to the NPR-C dimer (indicated in green and cyan with the protein surface shown in grey and light green). The sacubitril molecule fits into a hydrophobic region of NPR-C formed by Tyr140, Phe 164 and Tyr171. The acidic carboxylated groups possibly engage in hydrogen bonds with Arg166 and Arg211 of NPR-C, the amid group of saubitril forms another hydrogen bond with the carbonyl group of Lys209. The hydrophobic pocket corresponds to the binding site of Methionine 17 in CNP

## Discussion

The objective of the present study was to further elucidate the mechanisms underlying the direct cellular antiarrhythmic effects of sacubitrilat and the possible involvement of natriuretic peptide dependent signalling to identify new targets for future antiarrhythmic therapies in HF.

The following novel key findings can be derived: (i) CNP but not ANP exerts potent antiarrhythmic effects in a murine *in vitro* model of ion disbalance and increased diastolic Ca^2+^ leak mimicking HF, (ii) the antiarrhythmic effects of CNP can be inhibited through NPR-C-blockade, (iii) NPR-C activation alone yields similar antiarrhythmic effects as CNP-stimulation, (iv) the antiarrhythmic effects of sacubitrilat are not cGMP-mediated and can also be inhibited through NPR-C-blockade, and (v) sacubitrilat potently reduces SR-Ca^2+^ leak in a human iPSC-CM derived model of arrhythmia induced cardiomyopathy (AIC) through NPR-C.

To the best of our knowledge, this is the first study that identifies direct NPR-C activation as potential explanation for the antiarrhythmic effects of sacubitrilat as observed in large clinical trials.^9,10^ We have previously demonstrated that sacubitrilat, but not valsartan, exerts direct cellular antiarrhythmic effects in murine cardiomyocytes following isoproterenol stimulation as well as in isolated human cardiomyocytes from patients with heart failure, without compromising systolic Ca^2+^ release, sarcoplasmic reticulum Ca^2+^ content, or contractility.^16^ In addition, experimental studies have shown that sacubitrilat directly modulates cardiac electrophysiology by shortening action potential duration while reducing susceptibility to ventricular and atrial arrhythmias in isolated heart models.^37,38^ However, the cellular and molecular mechanisms underlying these beneficial antiarrhythmic effects have remained elusive.

Diastolic SR Ca^2+^ leak is recognized as a key contributor to arrhythmogenesis in AIC and other forms of heart failure, as it significantly disrupts the electrical stability of the heart.^2–4^ In our study, diastolic Ca^2+^ leak was experimentally induced by (i) enhancing Late I_NA_ in murine ventricular cardiomyocytes with ATX-II and (ii) tachyarrhythmic stimulation of iPSC-CM for 24 h.^5^ Elevated late I_NA_ is a characteristic pathophysiologic feature of heart failure, that prolongs action potential duration, facilitates the generation of afterdepolarizations, and enhances CaMKII/PKA signalling and thereby further augmenting proarrhythmogenic diastolic SR-Ca^2+^ leak.^4–8^ Through these combined mechanisms, enhanced late I_NA_ promotes ventricular arrhythmogenesis.^8^ In addition to these negative effects, CaMKII/PKA activation is known to increase SERCA2a activity through phosphorylation of phospholamban (PLN). Consistent with this, our data additionally show that the rate constant reflecting SERCA2a activity is increased upon treatment with ATX-II. This effect is still observed when ATX-II is combined with CNP or sacubitrilat, suggesting that the antiarrhythmic effects of CNP/NPR-C signalling do not negatively affect SERCA2a activity and lusitropy. Of note, Ca^2+^ elimination kinetics (RT50) of systolic Ca^2+^ transients are only improved/accelerated when ATX-II treatment is combined with CNP or sacubitrilat, as the high diastolic leak otherwise would impede Ca^2+^-elimination.

In this work, we show for the first time that sacubitrilat not only directly reduces arrhythmogenic SR-Ca^2+^ leak in murine CMs but also in a model of human AIC in an NPR-C-dependent manner. Thus, NPR-C might be a promising target to treat ventricular arrhythmias in AIC and other forms of heart failure. While previously considered a mere clearance receptor, it is nowadays established that NPR-C plays an active role in intracellular signalling.^19^ Upon activation, NPR-C couples with Gi proteins that in turn inhibit adenylyl cyclase activity, which leads to a reduction of cAMP levels and subsequently of PKA activation.^19^ PKA phosphorylates a range of downstream targets, including RyR2 at Serine 2808.^3^ Thus, through the inhibition of adenylyl cyclase via NPR-C, sacubitrilat might indirectly reduce the phosphorylation of RyR2 and thereby reduce SR Ca^2+^ leak. Importantly, there were no substantial alterations in SR Ca^2+^ load or systolic Ca^2+^ transient amplitude in response to sacubitrilat treatment. These findings indicate that sacubitrilat does not indirectly reduce diastolic Ca^2+^ release by modulating SR Ca^2+^ load, but rather specifically targets SR Ca^2+^ leak. Accordingly, no negative inotropic effect undesirable for use in heart failure was detected.

One potential mechanism for the NPR-C-dependent effect of sacubitrilat might be the local elevation of CNP levels through the inhibition of neprilysin-dependent degradation and a subsequently increased auto-/paracrine CNP-dependent signalling. In that case, however, increased intracellular cGMP-levels would be expected due to increased production upon binding of CNP to GC-B. This, however, was not observed. Our findings are consistent with a possible activation of NPR-C signalling in response to sacubitrilat. To the best of our knowledge, there exists no evidence that sacubitrilat directly binds to NPR-C. Our in-silico modelling analyses suggested a possible binding site of sacubitrilat in a hydrophobic region located between the dimer interface of NPR-C overlapping with the CNP binding-site. However, although *in silico* modelling may provide complementary pharmacological and structural support for NPR-C as a plausible mediator of the observed antiarrhythmic effects, it does not allow a definitive conclusion regarding direct binding of sacubitrilat to NPR-C. Consequently, dedicated experimental binding studies would be required to further evaluate the hypothesis of a direct interaction between sacubitrilat and NPR-C. The observation that the NPR-C-mediated antiarrhythmic effects of CNP and sacubitrilat were not reproduced by ANP is intriguing, particularly given the reported similar affinity of ANP and CNP for NPR-C. However, natriuretic peptide signalling in cardiomyocytes likely reflects a complex interplay between the different natriuretic peptide receptors. ANP-mediated cardioprotective effects have been shown to be absent in GC-A−/− mice, indicating a predominant reliance on GC-A signalling.^39^ This suggests that, despite potential NPR-C activation, ANP-induced signalling is likely dominated by GC-A-mediated pathways. Simultaneous activation of GC-A and NPR-C, compared with activation of GC-B and NPR-C by CNP, can lead to significantly different net effects on downstream processes, which may mask NPR-C-dependent functional effects at the cellular level. Accordingly, while NPR-C activation by ANP may occur, it does not translate into measurable antiarrhythmic effects in our study. Further, ligand-specific activation of NPR-C by CNP may contribute to the observed functional differences.

Interestingly, a recent publication found an antiarrhythmic effect of CNP on ventricular cardiomyocytes *in vitro*, that was abolished upon inhibition of phosphodiesterase 2 (PDE2A).^40^ Furthermore, it was found that CNP stimulation elevated cGMP levels, lowered cAMP levels and decreased phosphorylation of Ca^2+^ handling proteins.^40^ The authors suggested that CNP/GC-B/cGMP signalling leads to an activation of PDE2A, which in turn degrades cAMP.^40^ This is especially interesting, as decreased cAMP levels are also an important downstream effect of NPR-C activation. Thus, both pathways might provide an antiarrhythmic effect through decreased cAMP levels and subsequently lowered PKA activation. The authors also conducted *in vivo* experiments on the antiarrhythmic effect of CNP in PDE2 knockout mice. Interestingly, there was still a remarkable antiarrhythmic effect of CNP,^40^ which may be potentially explained through an activation of NPR-C. Sacubitrilat did not elevate cardiomyocyte cGMP levels in our study, suggesting that PDE 2 activation did not participate in the antiarrhythmic effects of sacubitrilat.

Besides our study, there are no published data regarding the effect of NPR-C activation on ventricular arrhythmias. However, several studies reported a cardioprotective effect of NPR-C on atrial fibrillation due to reduced atrial fibrosis and electrical remodelling.^41,42^ Thus, NPR-C might be a promising target to treat both atrial and ventricular arrhythmias.

### Limitations

This study is subject to several limitations.

1. As the experiments were conducted on ventricular cardiomyocytes *in vitro*, the findings may not fully translate to *in vivo* settings.
2. A major limitation of the present study is that the proposed role of NPR-C is based on complementary pharmacological modulation rather than genetic loss-of-function approaches. Consequently, our findings should be interpreted as hypothesis-generating and do not establish NPR-C as the definitive mediator of sacubitrilat's antiarrhythmic effects. Future studies employing genetic NPR-C knock-out or knock-down models, together with comprehensive analyses of downstream signalling pathways, including cAMP/PKA signalling, RyR2 phosphorylation, CaMKII activation, and transcriptional responses, will be essential to establish causality and define the molecular mechanisms underlying these observations.
3. The molecular docking analysis suggests that sacubitrilat may directly interact with the NPR-C binding pocket and provides a structural rationale for our pharmacological findings. However, these *in silico* data do not constitute direct evidence of receptor binding and should therefore be regarded as hypothesis-generating. Definitive validation of this proposed interaction will require dedicated binding studies.
4. Another limitation of this study is the use of iPSC-CM, which, despite representing a valuable human experimental model, do not fully reproduce the structural, electrophysiological, and functional characteristics of mature adult cardiomyocytes. Therefore, the translational relevance of these findings should be interpreted with caution, and direct extrapolation to the clinical setting is limited. Future studies in adult human cardiac tissue and clinical investigations will be required to confirm the translational applicability of these results.

## Conclusions

Sacubitrilat exerts potent antiarrhythmic effects through NPR-C activation without compromising systolic Ca^2+^ release, SR Ca^2+^ storage, and Ca^2+^ elimination kinetics. NPR-C activation may thus be a promising target for the development of urgently needed new antiarrhythmic drugs that can be used safely and effectively in heart failure.

## Supplementary Material

xvag253_Supplementary_Data

## Declarations

### Data Availability

The data underlying this article will be shared on reasonable request to the corresponding author.

### Pre-registered Clinical Trial Number

None supplied.
