# Supplementary material for: Sacubitrilat exerts potent antiarrhythmic effects through activation of natriuretic peptide receptor-C
Source: ESC Heart Fail. 2026 Oct 16;13(5):xvag253. doi: 10.1093/eschf/xvag253 (PMC13674804; doi:10.1093/eschf/xvag253)
Supplement: Supplementary file 1 [file xvag253_supplementary_data.docx]

*Supplemental Figure 1: Quality control of differentiation into iPSC-CM with ventricular subtype.* (**A):** Analysis of CM number for independent differentiation batches (n=8). Cells were stained with the cardiac marker cTNT and analyzed by flow cytometry.
(**B):** Semiquantitative PCR for the ventricular marker MYL2 and the atrial marker NR2F2 expression in two independent differentiations of ventricular iPSC-CM.
(**C):** Representative immunofluorescence image showing typical CM morphology and sarcomeric organization. Sarcomeres were visualized by staining for α-actinin (Z-discs) and titin M8/M9 (M-lines), revealing a well-defined striated pattern. Scale bar = 50 µm

*Supplemental Table 1: Primers used for Semiquantitative PCR*

| **Gene** | **Primer sequence (5´- 3´)** | **Product size** |
| --- | --- | --- |
| GAPDH | Fwd: AGAGGCAGGGATGATGTTCT  Rev: TCTGCTGATGCCCCCATGTT | 258 bp |
| MYL2 | Fwd: GGCGAGTGAACGTGAAAAAT  Rev: CAGCATTTCCCGAACGTAAT | 200 bp |
| NR2F2 | Fwd: CCGACCGGGTGGTCGCCTTTATGGA  Rev: CGGCTGGTTGGGGTACTGGCTCCTA | 226 bp |

*Supplemental Table 2: Antiodies used for immunofluorescence imaging*

| **Antibody** | **Company** | **RRID** |
| --- | --- | --- |
| cardiac troponin T (cTNT) | Miltenyi |  |
| Mouse-anti-α-actinin | Sigma | RRID: AB_476766 |
| Rabbit-anti-titin M8/M9 | Myomedix | RRID:AB_2734750 |
| Secondary antibody | | |
| AF488 donkey anti-mouse IgG | Invitrogen | RRID AB_162543 |
| AF555 donkey anti-rabbit IgG | Invitrogen | RRID AB_162543 |
| Nucleus staining | | |
| Hoechst | Sigma; #33258 | n.a. |
